# Laparoscopic appendectomy as the gold standard: What role remains for open surgery, conversion, and disease severity? An analysis of 32,000 cases with appendicitis in Germany

**DOI:** 10.1186/s13017-025-00626-2

**Published:** 2025-06-18

**Authors:** Claus Schildberg, Ulrike Weber, Volker König, Marius Linnartz, Sophie Heisler, Jennifer Hafkesbrink, Marcia Fricke, René Mantke

**Affiliations:** 1 Department of Surgery, Brandenburg Medical School Theodor Fontane, University Hospital Brandenburg/Havel, Hochstr. 29, Brandenburg, 14770 Germany; 2 Clinotel Hospital Association, 51149 Cologne, Germany; 3 Faculty of Health Science Brandenburg, Brandenburg Medical School Theodor Fontane, University Hospital Brandenburg/Havel, 14770 Brandenburg, Germany

**Keywords:** Appendectomy, Acute appendicitis, Laparoscopic appendectomy, Gold standard

## Abstract

**Background:**

Acute appendicitis is a common abdominal surgical emergency and is a major cause of acute abdomen in more than 20% of cases. Although various studies have been conducted in recent years on topics such as surgical techniques and antibiotic treatment of appendicitis, today there is a lack of large-scale studies focused on the different severity levels of acute appendicitis and their management. The study aimed to analyze the severity, types of surgical techniques, and mortality associated with acute appendicitis to identify possible developments.

**Methods:**

We conducted a retrospective multicenter observational study based on routine data from 2010 to 2022. Patients over 18 years old with acute appendicitis were included and the following data were collected: patient demographics, comorbidities, type of surgery, complications, admission to ICU, length of stay, and in-hospital mortality. A total of 31,988 patients were included in the study.

**Results:**

At the end of the study, 97.0% (*P* < .001) of the patients underwent laparoscopic appendectomy, with 86% of cases involving closure of the appendix stump by stapler (*P* < .001). It was only from 2014 onwards that more than 90% of surgeries were performed laparoscopically, and from 2017, this figure rose to 95%. Complicated appendicitis was present in 27.4% of cases. The distribution of severity was as follows: unspecified acute appendicitis in 39.5%, appendicitis with local peritonitis in 33.1%, appendicitis with local peritonitis and perforation in 17.1%, appendicitis with peritoneal abscess in 5.4%, and appendicitis with generalized peritonitis in 4.9%. Women had a significantly lower risk for conversion to an open operation than men (*P* < .001). The highest morbidity was observed in the group that converted from laparoscopy to open surgery (*P* <.001). Non-surgical treatment of appendicitis was not relevant, accounting for only 4% of cases.

**Conclusion:**

Since 2017, primary laparoscopic appendectomy has been the gold standard for even complicated acute appendicitis (> 95% annually). Over three-quarters of patients undergo an appendectomy with a stapler, making this surgical technique the preferred method of laparoscopic surgery in Germany. Patients who undergo an interoperative switch to open therapy should be considered a subgroup at risk of increased mortality.

**Trial registration:**

ClinicalTrials.gov ID: NCT06558760.

**Supplementary Information:**

The online version contains supplementary material available at 10.1186/s13017-025-00626-2.

## Introduction

Acute appendicitis is a common surgical abdominal emergency and one of the main causes of an acute abdomen in more than 20% of cases [1–3]. Laparoscopic or open appendectomy is the treatment of choice in most cases and therefore one of the most common visceral surgical emergency operations. The lifetime incidence of acute appendicitis is 8% with a peak occurrence between 20 and 40 years of age [3]. In recent years, some studies have addressed the outcomes and risk factors of morbidity after appendectomy [2, 4]. Additionally, scoring systems, radiological diagnostics, and the surgical management of acute, complicated, or uncomplicated appendicitis, as well as the timing of surgery, have been analyzed [5–12]. However, there is a lack of recent studies that analyze in detail the differences in the surgical treatment of various stages of appendicitis and their outcomes across a large number of cases. This study analyses the different stages of appendicitis, the development of laparoscopic and open approaches for appendectomy, the reasons for conversion, and in-hospital mortality using routine data from 68 hospitals in Germany between 2010 and 2022 (ClinicalTrials.gov ID: NCT06558760).

## Methods

The study is a retrospective multicenter observational study based on routine data from 2010 to 2022. The reporting follows the recommendations of the RECORD statement [13].

The study was conducted using routine administrative data from health insurance funds collected in accordance with § 301 SGB V and § 21 “Krankenhausentgeltgesetz (KHEntG)”.

The data was provided by the Clinotel Hospital Association Germany and derived from anonymized billing data according to the German Diagnosis-Related Groups (DRG) system for inpatient hospital treatments. The Clinotel Hospital Association Germany is a hospital association that includes small municipal, large municipal, district, and regional/university teaching hospitals with general surgery departments in Germany. Diagnoses were coded according to the International Classification of Diseases, German Modification, Coding Guideline ICD 10 GM [14]. Procedures were documented based on the German version of the International Classification of Procedures in Medicine (OPS) [15]. Secondary diagnoses/comorbidities were grouped according to Quan-Elixhauser at the patient level [16].

In the first step, we identified patients aged 18 years and over, diagnosed with acute appendicitis (ICD K35.x) between 2010 and 2022. In the subsequent step, we excluded cases without an appendectomy OPS code, those with nonspecific appendectomy OPS codes (OPS code 5.470.x or 5.470.y), or those who underwent appendectomy as part of another procedure. Furthermore, all cases with multiple appendectomy OPS codes were removed from the sample. Two cases with gender-neutral information on biological sex could not be included due to data protection regulations.

The following data was collected for analysis: patient demographics (biological sex, age), principle diagnosis of acute appendicitis ICD-10-GM K35.x, comorbidity conditions grouped by Quan-Elixhauser comorbidity, performed appendectomy (OPS code 5-470.0: open, OPS code 5-470.1: laparoscopic, OPS code 5-470.10: laparoscopic, closing with loop, OPS code 5-470.11: laparoscopic, closing with stapler, OPS code 5-470.2: conversion from laparoscopic to open) as well as postoperative outcome parameters, such as postoperative complications, admission to ICU, length of stay, and in-hospital-mortality. All OPS and ICD-10-GM codes used in this study are listed in the Supplement (eTable 1).

### Statistical analysis

Statistical analyses were performed using IBM SPSS Statistics (Version 25). Variables were summarized using absolute and relative frequencies for categorical parameters and means (SD) for continuous parameters. Comparisons were made using either the Fisher exact test, the χ2 test, or the Kruskal-Wallis test. All tests were used to compare the three groups as part of the overall analysis. To evaluate possible risk factors for conversion and in-hospital mortality, multivariable logistic regression with backward elimination (likelihood-ratio) was performed. The statistical significance level was set to 0.05 for all analyses. Since the study has an exploratory character, the P-values were used descriptively.

## Results

We identified 33,617 inpatients over the age of 18 years with a diagnosis of acute appendicitis (ICD K35.x) between 2010 and 2022. Among these, 1,458 cases (4.34%, 2010: 3.6%, 2022: 4.4%) involved acute appendicitis without any specific operative procedure. However, it is not possible to differentiate between patients who received conservative (antibiotic) therapy and those whose general condition was too poor for surgery using administrative data. The mortality in this group was significantly higher at 0.96% (*n* = 14) compared to the surgical group (0.14%, *n* = 47) analyzed subsequently. For the final analyses, 1613 cases without an appendectomy OPS code or with an appendectomy performed as part of another procedure were excluded, along with 3 cases with unspecific appendectomy OPS codes (OPS code 5.470.x or 5.470.y). Additionally, 11 cases with multiple appendectomy OPS codes were removed from the sample. Two cases with gender-neutral information on biological sex could not be included due to data protection reasons. As a result, 31,988 patients treated in 68 hospitals were available for the final analyses.

### Operation type and conversion rate according to appendicitis diagnosis

**Fig. 1:**
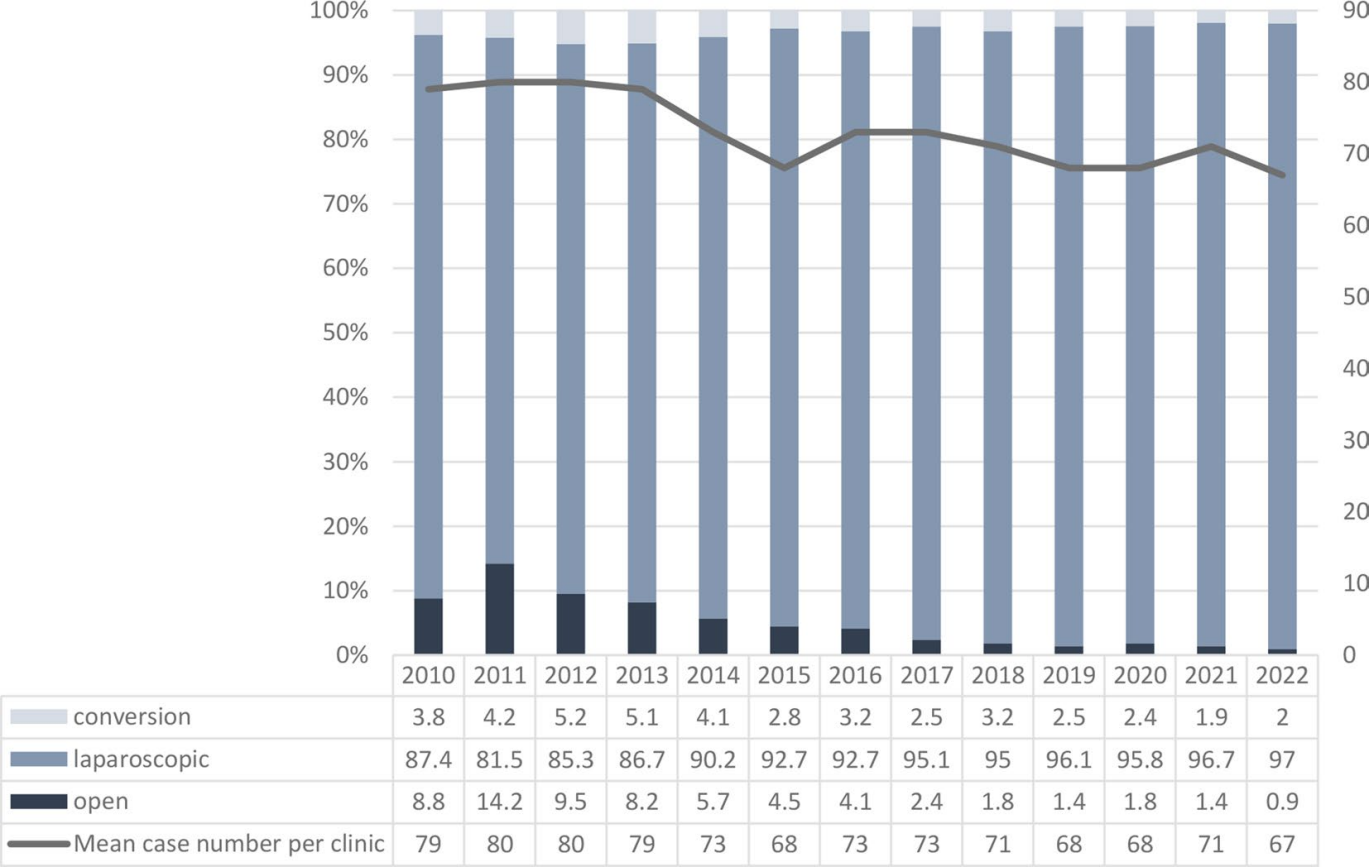
Operation Type and Conversion Rate (%), 2010–2022, *n* = 31 988

The proportion of laparoscopic appendectomies increased significantly from 87.4% in 2010 to 97% in 2022 (*P* <.001). The conversion rate from laparoscopic to open appendectomy remained nearly the same (Fig. 1).

**Fig. 2:**
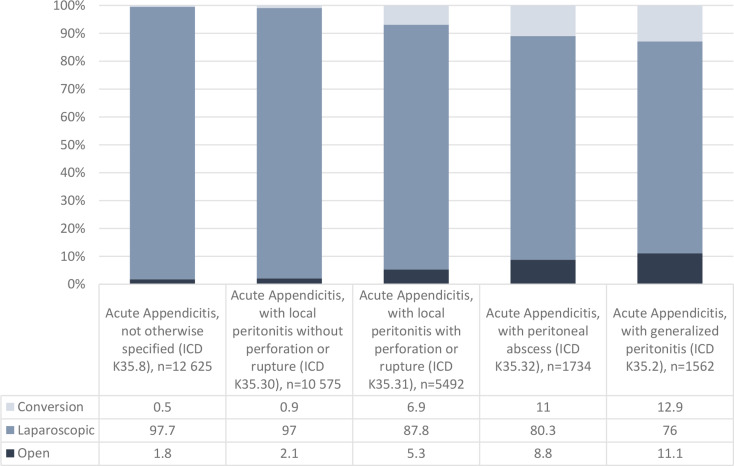
Operation Type according to Appendicitis Diagnosis (%), *n* = 31 988

As the severity of acute appendicitis increased, the proportion of laparoscopic operations decreased significantly. Appendicitis with local peritonitis was operated on laparoscopically in 97.0% of cases, while appendicitis with generalized peritonitis was operated on laparoscopically in 76% of cases (*P* <.001; Fig. 2).

The proportion of different appendicitis diagnoses remained nearly the same over the observation period (eFig. 1). Appendix removal by stapler increased from 74.1% in 2011 to 85.6% in 2022 (eFig. 2; *P* <.001).

### Patient characteristics, severity of appendicitis, and outcomes according to operation type

Of the patients, 16,582 patients (51.8%) were men, and 15,406 (48.2%) were women. The mean age was 41.3 years (SD, 17.9). The most common type of appendicitis was acute appendicitis not otherwise specified (ICD K35.8), affecting 12,625 patients (39.5%), followed by acute appendicitis with local peritonitis without perforation or rupture (ICD K35.30) in 10,575 patients (33.1%) (Table 1). More complicated forms of acute appendicitis accounted for 27.4% (*n* = 8788) of all cases: 17.1% (*n* = 5492) with local peritonitis and perforation or rupture (ICD K35.31), 5.4% (*n* = 1734) with local peritonitis and peritoneal abscess (ICD K35.32), and 4.9% (*n* = 1562) with generalized peritonitis (ICD K35.2). Regarding the type of appendicitis, 83.8% (*n* = 773) of the conversion group, 57.4% (*n* = 616) of the open surgery group, and 24.6% (*n* = 7399) of the laparoscopic group had more complicated forms of acute appendicitis (ICD K35.31, ICD K35.32, ICD K35.2)(*P* <.001) (Table 1). At least one postoperative surgical complication occurred in 1282 patients (4.0% of all patients): 20.8% (*n* = 192) of the conversion group, 17.3% (*n* = 186) of the open surgery group, and only 3.0% (*n* = 904) of the laparoscopic group had at least one postoperative complication (*P* <.001). Admission to ICU was necessary for 1,480 patients (4.4% of all patients) respectively for 13.0% (*n* = 121) of the conversion group, 8.0% (*n* = 87) of the open surgery group, and 4.0% (*n* = 1149) of the laparoscopic group *(P* < .001).

The overall in-hospital mortality was 0.1% (*n* = 47). The in-hospital mortality rate was highest in the open surgery group (open: 1.5% [*n* = 16], conversion: 1.3% [*n* = 12], laparoscopic: 0.1% [*n* = 19]).

**Table 1:** Patient characteristics and outcomes according to operation type

Characteristic	Total	Open	Laparoscopic	Conversion	*P* value
**No.** (%)	31 988	1073 (3.4)	29 992 (93.8)	923 (2.9)	
**Age**, mean (sd), y	41.3 (17.9)	52.8 (20.3)	40.5 (17.5)	54.2 (17.6)	< 0.001
**Sex**					
Female	15 406 (48.2)	421 (39.2)	14 642 (48.8)	343 (37.2)	< 0.001
Male	16 582 (51.8)	652 (60.8)	15 350 (51.2)	580 (62.8)	
**Uncomplicated acute appendicitis** (ICD K35.8, ICD K35.30**)**	23 200 (72.6)	457 (42.6)	22 593 (75.4)	150 (16.2)	< 0.001
**Complicated acute appendicitis** (ICD K35.2, ICD K35.31, ICD K35.32)	8788 (27.4)	616 (57.4)	7399 (24.6)	773 (83.8)	< 0.001
**Main diagnosis**					
Acute appendicitis, not otherwise specified (ICD K35.8)	12 625 (39.5)	237 (22.1)	12 333 (44.1)	55 (6.0)	< 0.001
Acute appendicitis with local peritonitis without perforation or rupture (ICD K35.30)	10 575 (33.1)	220 (20.5)	10 260 (34.2)	95 (10.3)	
Acute appendicitis with local peritonitis with perforation or rupture (ICD K35.31)	5492 (17.1)	291 (27.1)	4820 (16.1)	381 (41.3)	
Acute appendicitis with peritoneal abscess (ICD K35.32)	1734 (5.4)	152 (14.2)	1392 (4.6)	190 (20.6)	
Acute appendicitis with generalized peritonitis (ICD K35.2)	1562 (4.9)	173 (16.1)	1187 (4.0)	202 (21.9)	
**No. of secondary diagnoses** mean (sd)	4.6 (4.1)	4.9 (6.5)	1.9 (2.9)	5.2 (6.2)	< 0.001
**No. of secondary diagnoses**,** relevant for increasing remuneration**, mean (sd)	0.5 (1.1)	1.7 (2.7)	0.4 (0.9)	1.7 (2.5)	< 0.001
**Outcomes**					
**At least one of twelve examined complications (details in supplement)**	1282 (4.0)	186 (17.3)	904 (3.0)	192 (20.8)	< 0.001
**Admission to ICU**	1480 (4.4)	87 (8.0)	1149 (4.0)	121 (13.0)	< 0.001
**Mortality (in hospital)**	47 (0.1)	16 (1.5)	19 (0.1)	12 (1.3)	< 0.001
**Length of stay**, mean (sd), d	4.6 (4.1)	9.8 (10.1)	4.2 (3.0)	10.6 (9.7)	< 0.001

The average length of stay for all patients was 4.6 days. The mean length of hospital stay differed between the operation groups (conversion: 10.6 [SD, 9.7] days, open: 9.8 [SD, 10.1] days, laparoscopic: 4.2 [SD, 3.0] days; *P* <.001).

Patients undergoing an open operation or a conversion from laparoscopic to an open operation were significantly older than those undergoing a laparoscopic procedure (open: 52.8 [SD, 20.3] years, conversion: 54.2 [SD, 17.6] years, laparoscopic: 40.5 [SD, 17.5] years; *P* <.001). The proportion of men was highest in the conversion group (62.8%), followed by the open operation group with 60.8% men (laparoscopic: 51.2% men; *P* <.001,). Women have a significantly lower risk for conversion to an open operation than men (*P* <.001) (Table 1).

Most patients with acute appendicitis were aged between 18 and 29 years (*n* = 10898, 34.1%). The age distribution between men and women was nearly the same (eTable 2).

The proportion of different appendicitis diagnoses remained nearly the same over the observation period (eFig. 1).

The average number of secondary diagnoses was highest in the conversion group (conversion: 5.2 [SD, 6.2], open: 4.9 [SD, 6.5], laparoscopic 1.9 [SD, 2.9]; *P* <.001). Patients had an average of 4.6 secondary diagnoses (SD, 4.1). The most common secondary diagnosis was uncomplicated hypertension, present in 5,331 patients (16.7%) (eTable 3).

93.8% of operations (*n* = 29,992) were performed laparoscopically. More detailed information on the different complications according to the operation types can be found in the supplement (eTable 4).

### Factors for conversion and in-hospital mortality

The multivariable analysis identified the following variables associated with conversion: increasing age (OR, 1.011; 95%CI, 1.007–1.016; *P* <.001), sex (female vs. male: OR, 0.668; 95%CI, 0.579–0.770; *P* <.001), more complicated forms of acute appendicitis, and some secondary diagnosis (Table 2). Women have a significantly lower OR for conversion to an open operation than men (*p* <.001).

In order to clarify whether the conversion rates were related to age and severity of the appendicitis, a supplementary query was carried out. The analysis revealed an association between age and conversion rates for all appendicitis diagnoses, with higher age groups showing higher conversion rates. An association was also found between the severity of appendicitis and conversion rates in each age group, with more severe cases having nearly almost higher conversion rates (eFig. 3).

**Table 2:** Multivariable logistic regression associated with conversion laparoscopic to open, *n* = 30 915

Variable	OR (95% CI)	*P* value	
**Age**,** y**	1.011 (1.007–1.016)	< 0.001	
**Sex**,** female vs. male**	0.668 (0.579–0.770)	< 0.001	
**Diagnosis Acute Appendicitis**,** ICD 10**			
with generalized peritonitis vs. not otherwise specified	25.146 (18.352–34.456)	< 0.001	
with local peritonitis without perforation or rupture vs. not otherwise specified	1.855 (1.328–2.591)	< 0.001	
appendicitis with local peritonitis with perforation or ruptured vs. not otherwise specified	12.323 (9.185–16.533)	< 0.001	
with peritoneal abscess vs. not otherwise specified	21.450 (15.669–29.363)	< 0.001	
**Secondary Diagnosis**,** yes vs. no**			
Coagulopathy	1.605 (1.128–2.282)	< 0.01	
Diabetes, complicated	1.385 (1.068–1.795)	< 0.05	
Electrolyte disorders	1.604 (1.349–1.907)	< 0.001	
Other neurological disorders	1.815 (1.175–2.803)	< 0.01	
Obesity	1.320 (1.058–1.647)	< 0.05	
Peripheral vascular disorders	1.798 (1.192–2.712)	< 0.01	
Renal failure	1.510 (1.125–2.026)	< 0.01	

In the multivariable analysis regarding in-hospital-mortality, the following factors were identified as possible risk factors: increasing age (OR, 1.082; 95%CI, 1.055–1.110; *P* <.001), acute appendicitis with generalized peritonitis (ICD K35.2) (OR, 2.485; 95%CI, 1.280–4.823; *P* <.01), congestive heart failure (OR, 1.605; 95%CI, 1.128–2.282; *P* <.001), neurological disorders (OR, 1.815; 95%CI, 1.175–2.803; *P* <.001) and stab-/lacerated wounds (injury to blood vessel or abdominal organ, OR, 3.752; 95%CI, 1.869–7.534; *P* <.001). Furthermore, the type of operation is associated with in-hospital mortality (laparoscopy yes vs. no: OR, 0.259; 95%CI, 0.132 − 0.507; *P* <.001, Table 3).

**Table 3:** Multivariable logistic regression associated with In-Hospital-Mortality, *n* = 31 988

Variable	OR (95% CI)	*P* value	
Age, y	1.082 (1.055–1.110)	< 0.001	
Diagnosis: Acute Appendicitis with generalized peritonitis, yes vs. no	2.485 (1.280–4.823)	< 0.01	
Secondary Diagnosis, yes vs. no			
Congestive heart failure	1.605 (1.128–2.282)	< 0.001	
Other neurological disorders	1.815 (1.175–2.803)	< 0.001	
Procedure: Laparoscopy, yes vs. no	0.259 (0.132–0.507)	< 0.001	
Complication: Accidental stab-/lacerated wound according to surgery (Injury to blood vessel or abdominal organ), yes vs. no	3.752 (1.869–7.534)	< 0.001	

## Discussion

For many years, colleagues around the world believed that everything had been said about the therapeutic algorithms for treating appendicitis. However, this is only the case at first glance. The various manifestations of appendicitis and their treatment methods are by no means fully discussed. For example, there is currently debate about when uncomplicated acute appendicitis can be treated conservatively with antibiotics and painkillers without worsening outcomes [3, 17, 18]. We found that only a small portion of appendicitis cases admitted to hospital, 4.34%, were treated non-surgically, with an increase from 3.6% in 2010 to 4.4% in 2022.

There are also several studies on the value of open vs. laparoscopic surgery [19–33]. To have such discussions, the surgical results for the treatment of acute appendicitis must be clearly analyzed and published up to date. For this purpose, we used routine administrative data from health insurance companies collected from patients in the Clinotel hospital network in Germany. The aim was to use the data collected from 33,000 patients to identify possible peculiarities in the patient characteristics, treatment, and therapy of acute appendicitis.

The age and gender distribution data we collected showed that appendicitis occurs primarily at young ages; 67% of cases occurred in patients aged 49 years and younger. The gender distribution was almost equal.

Almost three-quarters of the cases (72.6%) were uncomplicated appendicitis.

This is consistent with the results of other research groups in the literature review [34, 35].

Our results show an almost complete conversion to laparoscopic appendectomy. The proportion of laparoscopic appendectomies increased significantly from 87.4% in 2010 to 97.0% in 2022 (*P* <.001). Appendix removal by stapler increased from 74.1% in 2011 to 85.6% in 2022. Usually the surgeon decides how the appendix base will be closed. In some hospitals in Germany the stapler is generally used. If the base of the appendix is not relevantly involved in the inflammation (uncomplicated appendicitis), discontinuation with a double Roeder loop seems also be safe [36, 37]. However, the data we use does not provide information about the reasons for the use of the procedure (stapler vs. Roeder loop).

In our opinion, open appendectomy no longer plays a significant role when considering the entire operated group; the laparoscopic approach has become the gold standard for all stages of appendicitis.

Comparing our results with the publications of other groups, the situation is not so clear. Thompson et al. reported an open surgery rate of 4.8% in 3019 patients studied, which is 2.5 times higher than in our group [38]. In another publication with over 65,000 patients from 2013, the proportion of laparoscopic procedures was only 33.8% [39]. All these results show that our study demonstrates a nearly complete conversion to laparoscopic surgery for the first time. This development took several decades. This is not surprising, as there are already theories about the introduction and spread of technologies in this context. The diffusion of an innovation involves five phases: adoption by innovators, followed by early adopters, the early majority, the late majority, and finally the laggards [40].

In addition, good ideas must be expressed through conversations (“word of mouth”). The tipping point in favor of surgical technology in this case is an average acceptance of this procedure by 20% of the users (e.g. surgeons). This clearly shows why it takes so long for a procedure to spread [41–44]. According to our study, it took 37 years from the first laparoscopic appendectomy in 1980 to achieve a continuous laparoscopic rate of over 95%.

This raises the question: How innovative is surgery today? It is time to discuss this, especially with a diagnosis as common as acute appendicitis. From our perspective, surgeons must approach such processes more constructively. In addition to the above-mentioned descriptive theories on the dissemination of innovations, we need better involvement of our professional representatives such as the surgical society, which promotes the dissemination of innovations In our opinion, the surgical societies should play a more important role in this process. Significant surgical innovations should not be left to chance or the market; this is ethically problematic. We argue that the widespread implementation of an innovation with such clear advantages as laparoscopic appendectomy should take no longer than 12–15 years. How can this be achieved? We suggest that surgical societies form innovation teams. They could rank important projects and studies from congresses or publications several times a year to identify relevant innovations. The focus should be on projects that could either relatively improve the therapy of a large group of patients or greatly improve the therapy of a smaller group of patients. The innovation teams should then develop a plan for the innovations, demonstrating the potential of new methods. This includes, for example, study planning and benefit/cost analysis. When it comes to important potential innovations, it should not be left to chance how intensively and with what resources they are pursued. As a rule, when innovations are introduced, only case studies are available, possibly in comparison with retrospective data. The innovation teams would then need to verify the results through multi-center analyses or, ideally, prospective randomized studies to promptly incorporate them into guidelines. We believe that the financing of these processes should not be left to health insurance companies or medical companies, as there is a risk of conflicts of interest on both sides. In addition to third-party funding, state funds should also be available for these processes, as the state must have an interest in promoting innovations for its citizens.

Our data concerning the surgical complication rate of 4%, intensive care unit admission of 4.4%, and overall in-hospital mortality of 0.1% (total population) is consistent with publications from other groups [45–49].

Our results showed that patients who underwent open surgery or conversion from laparoscopic to open surgery were significantly older than those who underwent laparoscopic surgery (*P* <.001). The proportion of men was highest in the conversion group (62.8%), followed by the open surgery group with 60.8% men (laparoscopic: 51.2% men; *P* <.001), indicating that women have a significantly lower risk for conversion to an open operation than men (*p* <.001). 13.0% (*n* = 121) of the conversion group required admission to the intensive care unit (open: 8.0% (*n* = 87), laparoscopic: 4.0% (*n* = 1149); *P* <.001). Accordingly, the average length of hospital stay was different among the surgical groups (*P* <.001). The in-hospital mortality rate was highest in the open surgery group (*P* <.001). A comparison of our results with publications from other groups shows similar results [13, 50–54].

The multivariable analyses of our cohort identified the following variables associated with conversion: increasing age (*P* <.001), gender (female vs. male: *P* <.001), and for in-hospital mortality, increasing age (*P* <.001) and occurrence of (a) acute appendicitis with generalized peritonitis (*P* <.001), (b) heart failure (*P* <.001), (c) neurological disorders (*P* <.001), and (d) a puncture/cut wound. In addition, the type of surgery is associated with in-hospital mortality (laparoscopy yes vs. no; *P* <.001).

Due to the size of the study, we were able to examine both the above-mentioned epidemiological data and clinical data in the multivariate analysis carried out.

Taking into account the newly published literature, this study was able to specify the risk parameters already known from other studies, such as age and gender, as well as complications and mortality. Additionally, our study identified neurological disorders as a risk factor [51, 55–57].

## Key results

- Tailoring therapy for appendicitis remains a challenge.
- Non operative therapy actually played no relevant role by inpatients in German hospitals.
- Laparoscopic stapler appendectomy is the most commonly performed surgery and is now the gold standard for all stages of appendicitis (> 95%).
- The primarily open approach has a niche existence.
- Conversion appendectomy, in particular, leads to significantly increased morbidity, which is associated with worse postoperative outcomes, and requires special follow-up care especially in elder patients.
- Women have a significantly lower risk for conversion to an open operation than men.

## Limitations

Because the analysis is based on administrative data collected primarily for billing purposes, it has the usual limitations of such data. This includes, above all, partially incomplete clinical data. So, in addition to the OPS and ICD-10 codes, no information on other clinical parameters e.g. intraoperative findings, imaging, antibiotic use are recorded in the data. However, the plausibility of the documented diagnoses and procedures is checked by the Clinotel hospital network and corrected if necessary before the data are transmitted to the health insurance companies for billing. It should also be noted that the hospital network mainly comprises medium-sized hospitals. However, since the surgical treatment of appendicitis is part of the standard repertoire of general surgery departments, no systematic bias with respect to case severity is to be expected.

## Electronic supplementary material

Below is the link to the electronic supplementary material.

Supplementary Material 1

## Data Availability

No datasets were generated or analysed during the current study.
