# Supplementary material for: Laparoscopic appendectomy as the gold standard: What role remains for open surgery, conversion, and disease severity? An analysis of 32,000 cases with appendicitis in Germany
Source: World J Emerg Surg. 2025 Jun 18;20:53. doi: 10.1186/s13017-025-00626-2 (PMC12178000; doi:10.1186/s13017-025-00626-2)

**eTable 1: Procedure Definitions**

**eFigure 1: Acute Appendicitis by Type of Appendicitis (ICD K35.x)**

**eFigure 2: Laparoscopic Procedures, Stapler vs. Loop**

**eTable 2: Age Distribution According to Sex**

**eTable 3: Postoperative Complications according to Operation Type**

**eTable 4: Secondary Diagnosis (Quan-Elixhauser) by Sex**

**eFigure 3: Conversionrate by Diagnosis and Age**

**eTable 1: Procedure Definitions**

| Definition | Condition | Procedure/Diagnosis codes |
| --- | --- | --- |
| Diagnosis |  |  |
|  | Acute appendicitis with generalized peritonitis | K35.2 |
|  | Acute appendicitis with local peritonitis without perforation or rupture | K35.30 |
|  | Acute appendicitis with local peritonitis with perforation or rupture | K35.31 |
|  | Acute appendicitis with peritoneal abscess | K35.32 |
|  | Acute appendicitis, not otherwise specified | K35.8 |
| OP-Procedure |  |  |
|  | Appendectomy, open | 5-470.0 |
|  | Appendectomy, laparoscopic | 5-470.1 |
|  | Appendectomy, laparoscopic, loop | 5-470.10 |
|  | Appendectomy, laparoscopic, stapler | 5-470.11 |
|  | Appendectomy, laparoscopic, other | 5-470.1x |
|  | Appendectomy, conversion laparoscopic to open | 5-470.2 |
| Complication |  |  |
|  | Abdominal wall closure | 5-545.0, 5-545.x or 5-545.y |
|  | Admission of red blood cell concentration | 8-800.c |
|  | Bleeding/Haematoma | T81.0 |
|  | Cystitis | N30.0 |
|  | Embolism/Thrombosis | I26, I80, I82.2, I 82.8 or I82.9 |
|  | Noscomial pneunomia | U69.00 or U69.01 |
|  | Postop. Infection | T81.4 |
|  | Postop. Intestinal obstruction | K91.3 |
|  | Relaparatomy | 5-983 |
|  | Rupture OP-wound | T81.3 |
|  | Secundary fissure | 5-467.0 |
|  | Stab-/Lacerated wound | T81.2 |

**eFigure 1: Acute Appendicitis by Type of Appendicitis (ICD K35.x)**


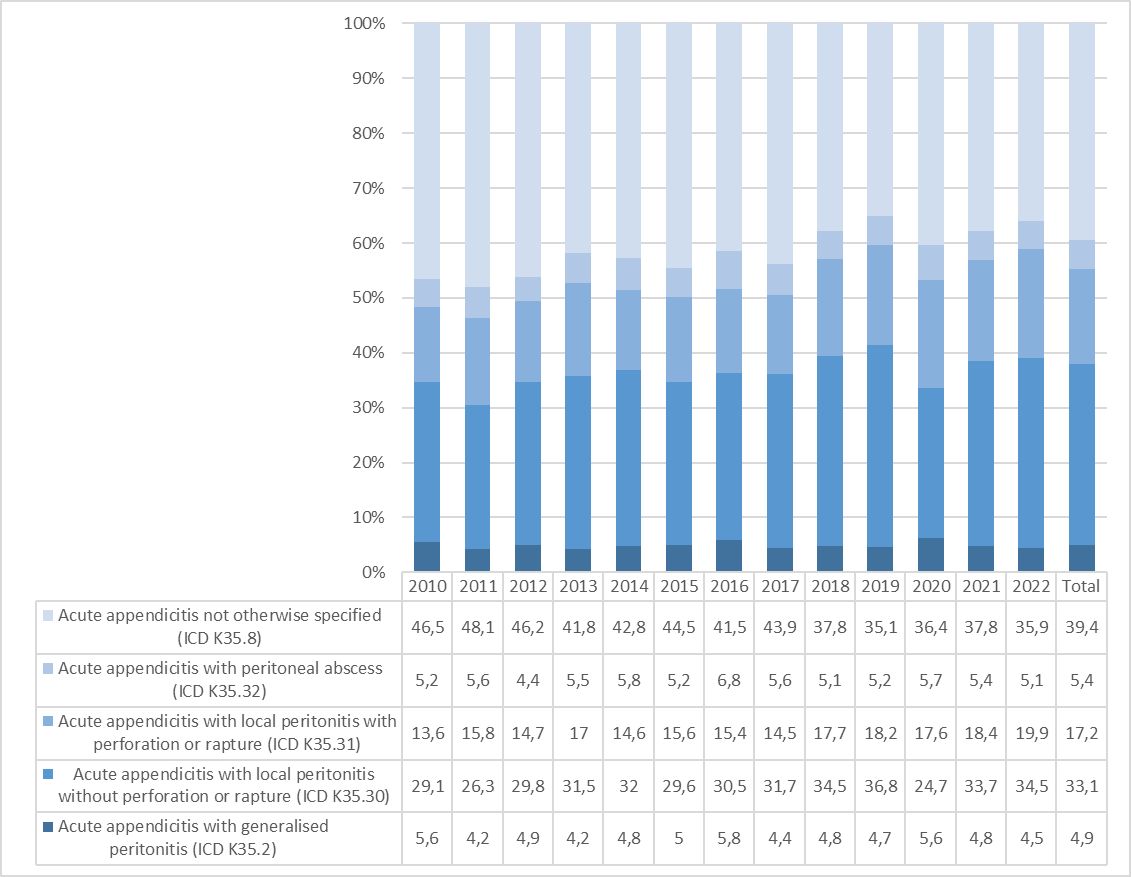


**eFigure 2: Laparoscopic Procedures, Stapler vs. Loop**

**eTable 2: Age Distribution According to Sex**

| **Characteristic** | **Total** | **Female** | **Male** | **P value** |
| --- | --- | --- | --- | --- |
| No. (%) | 31 988 | 15 406 (48.2) | 16 582 (51.8) |  |
| Age Group | | | | |
| 18-29 | 10 898 (34.1) | 5565 (36.1) | 5333 (32.2) | *.048* |
| 30-39 | 5974 (18.7) | 2654 (17.2) | 3320 (20.0) |  |
| 40-49 | 4527 (14.2) | 2034 (13.2) | 2493 (15.0) |  |
| 50-59 | 4921 (15.4) | 2425 (15.7) | 2496 (15.1) |  |
| 60-69 | 3067 (9.6) | 1478 (9.6) | 1589 (9.6) |  |
| 70-79 | 1745 (5.5) | 795 (5.2) | 950 (5.7) |  |
| 80-89 | 790 (2.5) | 415 (2.7) | 375 (2.3) |  |
| > 90 | 66 (0.2) | 40 (0.3) | 26 (0.2) |  |

**eTable 3: Secondary Diagnosis (Quan-Elixhauser) by Sex**

| **Characteristic** | **Total** | **Female** | **Male** | **P Value** |
| --- | --- | --- | --- | --- |
| No. (%) | 31 988 | 15 406 (48.2) | 16 582 (51.8) |  |
| Secondary Diagnosis | | | | |
| AIDS | * | * | * | - |
| Alcohol | 60 (0.2) | 17 (0.1) | 43 (0.3) | <.01 |
| Blood_loss_anemia | 12 (0.04) | * | * | <.01 |
| Deficiency_anemia | 76 (0.2) | 54 (0.4) | 22 (0.1) | <.001 |
| Cardiac_arrhythmias | 978 (3.1) | 381 (2.5) | 597 (3.6) | <.001 |
| Chronic_pulmonary_disease | 832 (2.6) | 382 (2.5) | 450 (2.7) | .18 |
| Coagulopathy | 413 (1.3) | 169 (1.1) | 244 (1.5) | <.01 |
| Congestive_heart_failure | 396 (1.2) | 172 (1.1) | 224 (1.5) | .06 |
| Depression | 607 (1.9) | 393 (2.6) | 214 (1.3) | <.001 |
| Diabetes_complicated | 151 (0.5) | 63 (0.4) | 88 (0.5) | .11 |
| Diabetes_uncomplicated | 1050 (3.3) | 389 (2.5) | 661 (4.0) | <.001 |
| Drug_abuse | 34 (0.1) | 7 (0.05) | 27 (0.2) | <.001 |
| Fluid_electrolyte_disorders | 2685 (8.4) | 1513 (9.8) | 1172 (7.1) | <.001 |
| Paralysis | 79 (0.3) | 23 (0.2) | 56 (0.4) | <.001 |
| Hypertension_uncomplicated | 5331 (16.7) | 2432 (15.8) | 2899 (17.5) | <.001 |
| Hypertension_complicated | 205 (0.6) | 92 (0.6) | 113 (0.4) | .35 |
| Hypothyroidism | 2186 (6.8) | 1671 (10.8) | 515 (3.1) | <.001 |
| Lymphoma | 11 (0.03) | * | * | .43 |
| Tumor_without_metastasis | 148 (0.5) | 74 (0.5) | 74 (0.4) | .65 |
| Metastasic_cancer | 32 (0.1) | 17 (0.1) | 15 (0.09) | .88 |
| Liver_disease | 313 (1.0) | 127 (0.8) | 186 (1.1) | <.01 |
| Other_neurological_disorders | 335 (1.0) | 161 (1.1) | 174 (1.0) | .97 |
| Obesity | 2201 (6.9) | 1053 (6.8) | 1148 (6.9) | .76 |
| Peptic_ulcer_without_bleeding | 5 (0.02) | * | * | .60 |
| Peripheral_vascular_disorders | 259 (0.8) | 86 (0.6) | 173 (1.0) | <.001 |
| Psychoses | 55 (0.2) | 17 (0.1) | 38 (0.2) | <.01 |
| Pulmonary_circulation_disorders | 53 (0.2) | 27 (0.2) | 26 (0.2) | .70 |
| Renal_failure | 613 (1.9) | 270 (1.2) | 343 (2.1) | <.05 |
| Rheumatoid_arthritis_vascular_diseases | 126 (0.4) | 87 (0.6) | 39 (0.2) | <.001 |
| Valvular_disease | 134 (0.4) | 52 (0.3) | 82 ((0.5) | <.05 |
| Weight_loss | 68 (0.2) | 38 (0.3) | 30 (0.2) | .20 |

*data protection (n<5)

**eTable 4: Postoperative Complications according to Operation Type**

| **Characteristic** | **Total** | **Open** | **Laparoscopic** | **Conversion** | | **P value** |
| --- | --- | --- | --- | --- | --- | --- |
| **No.** (%) | 31 988 | 1073 (3.4) | 29 992 (93.8) | 923 (2.8) |  | |
| **Complication** |  |  |  |  |  | |
| Abdominal wall closure | 54 (0.2) | 17 (1.6) | 15 (0.1) | 22 (2.4) | <.001 | |
| Admission of red blood cell concentration | 58 (0.2) | 10 (0.9) | 41 (0.1) | 7 (0.8) | <.001 | |
| Bleeding/Haematoma | 301 (0.9) | 26 (2.4) | 253 (0.8) | 22 (2.4) | <.001 | |
| Cystitis | 67 (0.2) | 6 (0.6) | 55 (0.2) | 6 (0.7) | <.001 | |
| Embolism/Thrombosis | 0 (0) | 0 (0) | 0 (0) | 0 (0) | - | |
| Noscomial pneunomia | 49 (0.2) | 6 (0.6) | 30 (0.1) | 13 (1.4) | <.001 | |
| Postop. Infection | 184 (0.6) | 15 (1.4) | 145 (0.5) | 24 (2,6) | <.001 | |
| Postop. Intestinal obstruction | 213 (0.7) | 29 (2.7) | 143 (0.5) | 41 (4.4) | <.001 | |
| Relaparatomy | 157 (0.5) | 7 (0.7) | 140 (0.5) | 10 (1.1) | <.05 | |
| Rupture wound | 78 (0.2) | 15 (1.4) | 33 (0.1) | 30 (3.3) | <.001 | |
| Secundary fissure | 32 (0.1) | * | 28 (0.1) | * | n.s | |
| Stab-/Lacerated wound | 687 (2.1) | 143 (13.3) | 422 (1.4) | 122 (13.2) | <.001 | |

*: data protection (n<5)

**eFigure 3: Conversionrate by Diagnosis and Age**


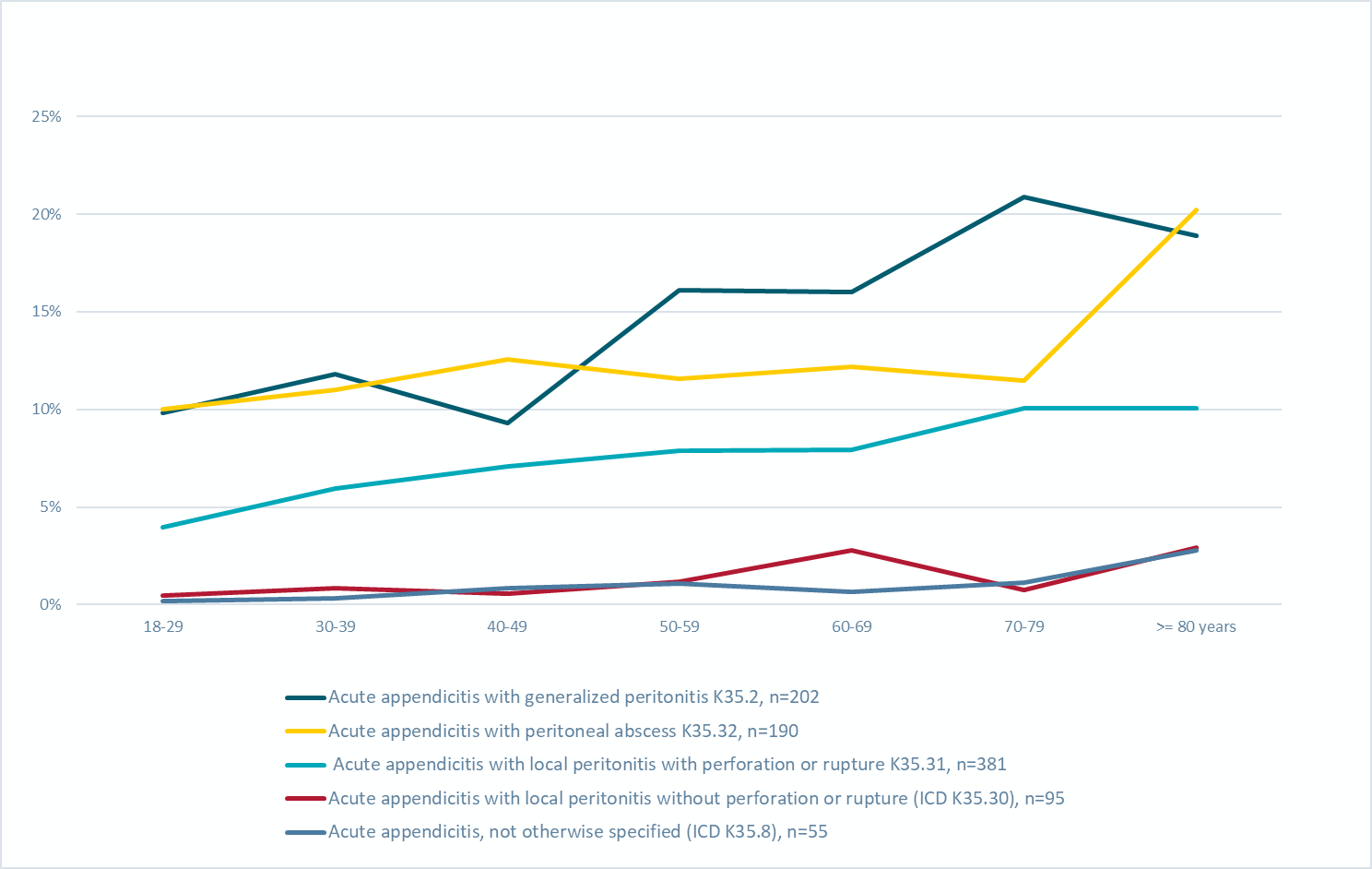

Supplement: Supplementary file 1 — Supplementary Material 1 [file 13017_2025_626_MOESM1_ESM.docx]
